# Sclerosing Mesenteritis: A Rare Cause of Abdominal Pain

**DOI:** 10.7759/cureus.28573

**Published:** 2022-08-30

**Authors:** Said Sharawi, Vincent Graffeo, Lynne J Goebel

**Affiliations:** 1 Internal Medicine, Marshall University Joan C. Edwards School of Medicine, Huntington, USA; 2 Pathology, Marshall University Joan C. Edwards School of Medicine, Huntington, USA

**Keywords:** fat necrosis, melanoma, lymphoma, abdominal pain, sclerosing mesenteritis

## Abstract

Sclerosing mesenteritis (SM) is a rare inflammatory fibrotic disease of the small intestine mesenteric fat often discovered incidentally on a CT scan. Clinical manifestations depend on the mass effect on the viscera and vessels. The most common symptoms are abdominal pain, bloating, and nausea. SM occurs predominantly in Caucasian men, during the fifth to seventh decades of life. We present a 69-year-old woman with SM whose symptoms were thought to be from irritable bowel syndrome.

A 69-year-old female with a history of fibromyalgia presented with recurrent bouts of abdominal pain across her mid-abdomen lasting 30 minutes to an hour associated with nausea, alternating constipation and diarrhea with occasional mucus, and bloating. She used bismuth subsalicylate and ondansetron with temporary relief. Upper endoscopy and colonoscopy were unrevealing. Initially, she was felt to have irritable bowel. Later she presented with nausea and right upper quadrant pain and underwent cholecystectomy. When her pain recurred, the patient had a CT abdomen and pelvis which showed multiple sub-centimeter mesenteric lymph nodes with surrounding haziness and stranding in the root of the mesentery consistent with SM. The patient had a pannus biopsy showing fat necrosis that confirmed the diagnosis. She continued to have waxing and waning symptoms over several years and in the interim was diagnosed with melanoma limited to the skin. The patient had a particularly severe episode of abdominal pain prompting a repeat CT scan with a subsequent biopsy of an enlarged left para-aortic lymph node that revealed lymphoma.

Our patient’s diagnosis of SM was delayed as her symptoms were mistaken for irritable bowel syndrome. Worsening symptoms should alert clinicians to an alternate diagnosis such as SM. There are characteristic radiographic findings on CT scans and biopsy of the lesions. SM’s association with neoplastic diseases such as lymphoma, melanoma, colorectal, and prostate cancer is controversial, however, practitioners should be aware of this possibility and consider biopsy for any suspicious lesions.

## Introduction

Sclerosing mesenteritis (SM) is a rare benign idiopathic inflammatory disorder that presents with fat necrosis and fibrosis in the mesentery of the small bowel [1]. A systematic review of 194 cases in the literature described the disease with different names including mesenteric panniculitis, retractile mesenteritis, idiopathic mesenteric fibrosis, misty mesentery, systemic nodular panniculitis, liposclerotic mesenteritis, mesenteric Weber-Christian disease, xanthogranulomatous mesenteritis, mesenteric lipogranuloma, and mesenteric lipodystrophy but used SM as the overarching name [2]. It is characterized by different pathologies including nonspecific chronic inflammation around the vessels, fibrosis, fat necrosis, and lipid-laden foamy macrophages [1].

SM is more common in men than women with a ratio of 2:1. It mostly occurs during the fifth to seventh decades of life, with a patient’s median age of 65 years [1]. Patients with SM mainly complain of chronic abdominal pain with other non-specific symptoms like bloating, nausea, vomiting, diarrhea, constipation, and weight loss [1]. The diagnosis can be delayed as the symptoms mimic other more common disorders. We present a 69-year-old woman with SM whose symptoms were thought to be from irritable bowel syndrome.

This case was presented at Marshall University Health Sciences Research Day on October 29, 2021.

## Case presentation

**Figure 1:**
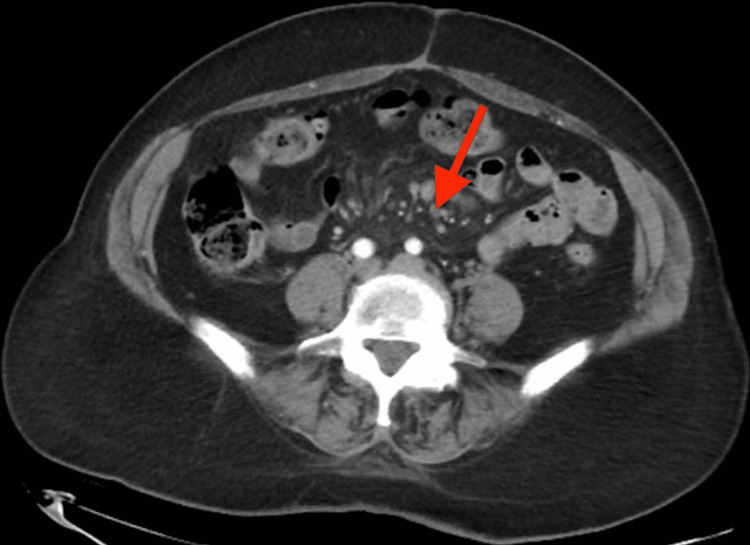
CT abdomen and pelvis with sub-centimeter lymph nodes and surrounding haziness and stranding in the root of the mesentery (red arrow)

A 69-year-old female with a history of fibromyalgia presented with recurrent bouts of abdominal pain across her mid-abdomen lasting 30 minutes to an hour associated with nausea, alternating constipation and diarrhea with occasional mucus, and bloating. Initially, she was felt to have irritable bowel syndrome. She used bismuth subsalicylate and ondansetron with temporary relief. Later she presented with nausea and right upper quadrant pain and was found to have cholecystitis, so she underwent cholecystectomy. When her pain recurred, the patient had a CT abdomen and pelvis which showed multiple sub-centimeter mesenteric lymph nodes with surrounding haziness and stranding in the root of the mesentery consistent with SM (Figure 1).

**Figure 2:**
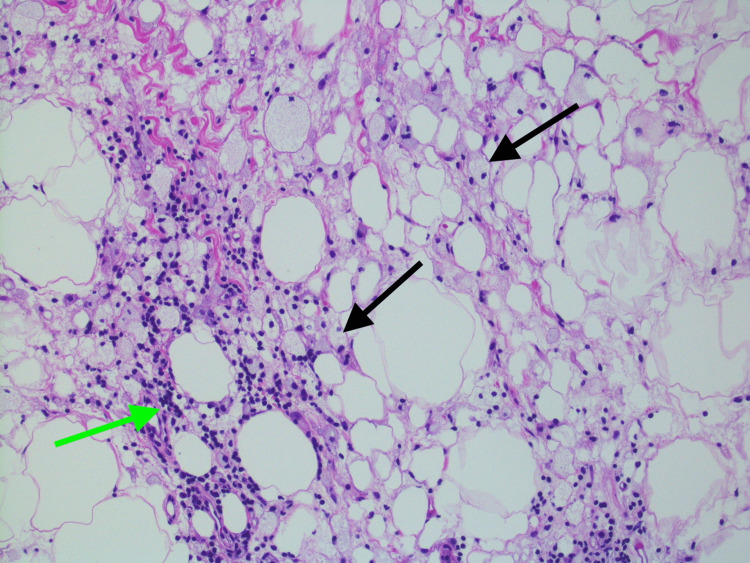
Mesenteric adipose tissue biopsy with fat necrosis, chronic inflammation (green arrow) and lipid-laden macrophages (black arrows), 100x magnification, hematoxylin and eosin staining.

**Figure 3:**
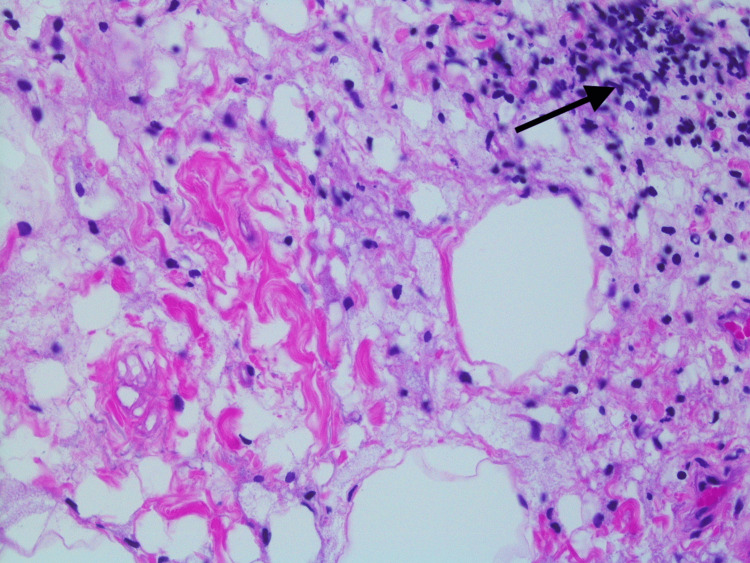
Mesenteric adipose tissue biopsy with fat necrosis and chronic inflammation (black arrow), 400x magnification, hematoxylin and eosin staining

**Figure 4:**
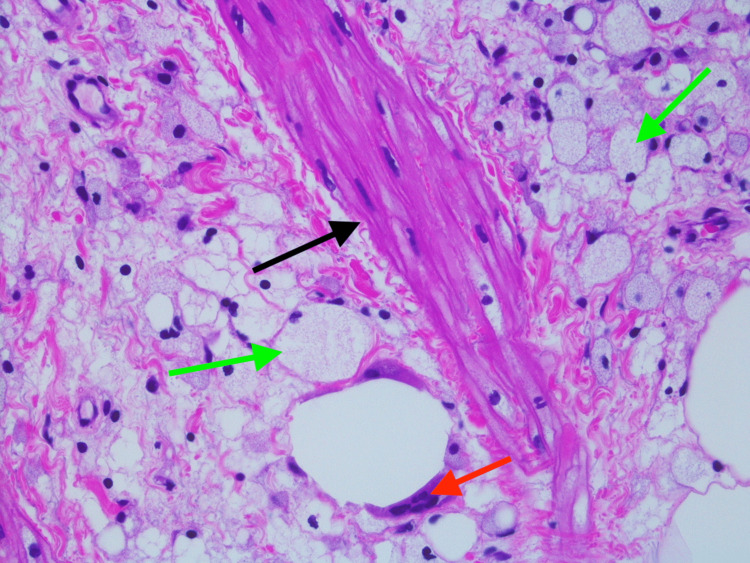
Mesenteric adipose tissue biopsy showing fibrous banding (black arrow) and fat necrosis with lipid-laden macrophages (green arrow) and multinucleated giant cells (red arrows), 400x magnification, hematoxylin and eosin staining

She was referred to a tertiary care hospital where she had an EGD and colonoscopy and another CT abdomen and pelvis with similar findings. Biopsy of the mesenteric adipose tissue showed fat necrosis with chronic inflammation, lipid-laden macrophages, and scattered collagen fibrous bands (Figures 2-4).

The patient went for several years with waxing and waning symptoms and in the meantime developed melanoma limited to the skin. Eventually, her symptoms of abdominal pain and nausea increased and she required admission to the hospital where another CT abdomen showed enlarged retroperitoneal lymph nodes with a left para-aortic node that measured 1.9 x 1.7 x 3.9 cm.

**Figure 5:**
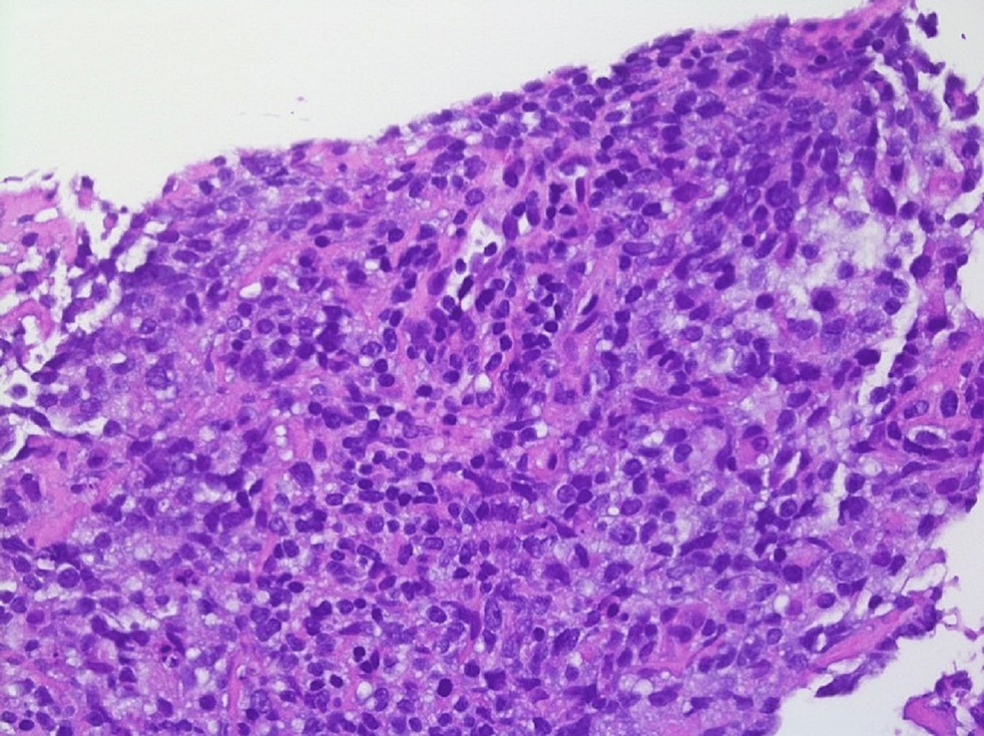
Hematoxylin and eosin staining of lymph node (400x power)

**Figure 6:**
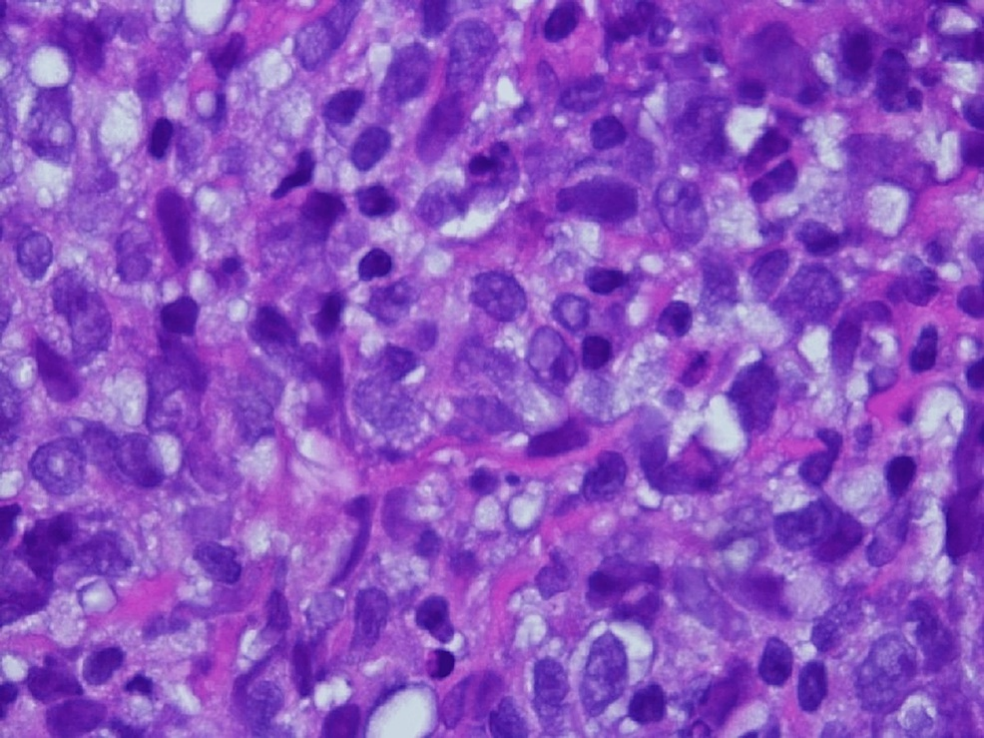
Hematoxylin and eosin staining of lymph node (1,000x power)

CT-guided biopsy of the left para-aortic lymph node with flow cytometry showed a neoplastic lymphoid proliferation. On microscopic examination, the tumor cells included smaller cells with twisted and angulated nuclei and many larger cells with centroblast morphology and scattered individual cell necrosis/apoptosis (Figures 5, 6).

**Figure 7:**
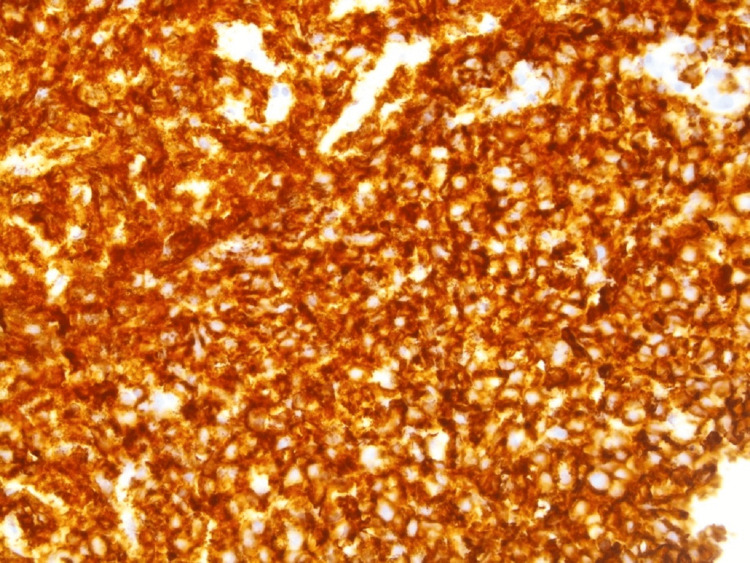
CD20 staining of lymph node (400x power)

Flow cytometry showed a monoclonal CD10-positive, lambda-positive B-cell population. The neoplastic lymphocytes were positive for CD20 (Figure 7).

**Figure 8:**
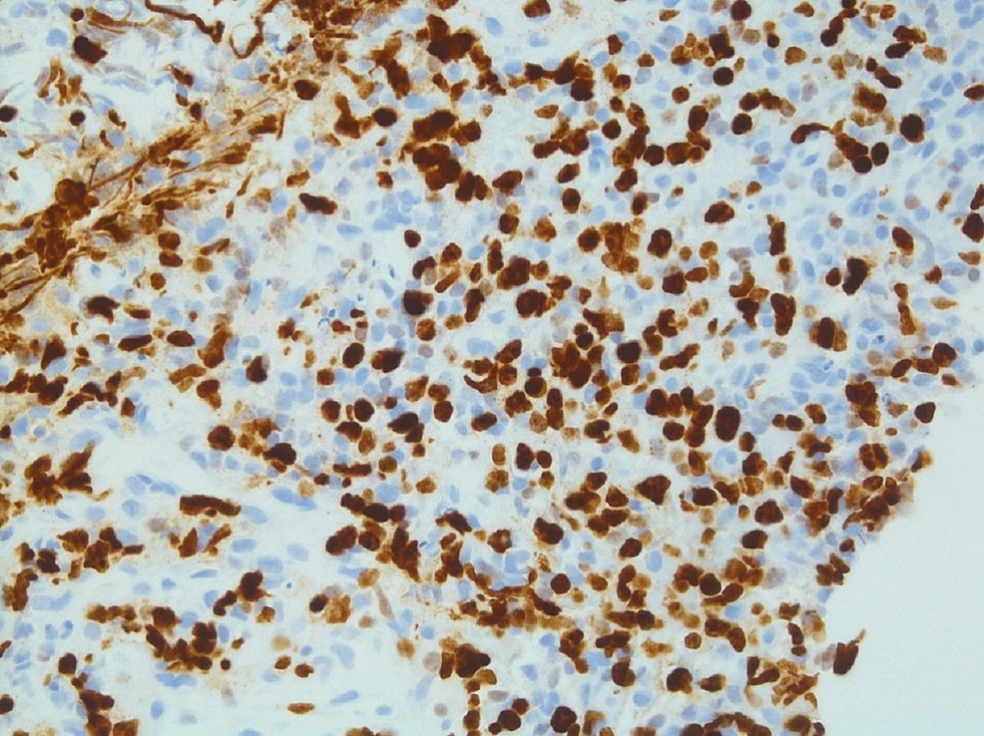
Ki-67 staining of lymph node (400x power)

FISH detected IGH/BCL2 translocation and rearrangement of BCL6. There was no evidence of MYC/IGH-specific gene translocation. Ki-67 labeling rate was about 25% (Figure 8).

MUM1 was positive and CD21 did not show preserved follicular dendritic cells. The differential diagnosis included an intermediate to higher-grade follicular lymphoma or diffuse large B-cell lymphoma.

## Discussion

Our patient presented with chronic abdominal pain that was initially thought to be irritable bowel syndrome, a much more common disease, and was later diagnosed with SM, a rare disease seen in 0.6% of over 7,000 consecutive patients having abdominal CT scans [3]. Our patient also had both melanoma and lymphoma. There are some reports of SM being a paraneoplastic syndrome but there is conflicting data. Sharma et al. only found that 8.9% of SM patients had cancer [2] and a case-controlled CT scan study by Gogebakan et al. showed no difference in cancers found between groups [4]. Similarly, Khasminsky et al. followed 166 patients with non-Hodgkin lymphoma in a case-control study for five years and found a 1.8% prevalence of SM among these patients which was not significantly different from controls [5]. On the other hand, Badet et al. reviewed CT scans of 158 patients with SM and found cancer in just over half of the cases, but there was no control group [6]. The most common cancers in this series were lymphoma, melanoma, colon, and prostate two of which were present in our patient. Kipfer et al. found lymphoma in 15% of their surgical case series [7]. A CT scan case-control study by van Putte-Katier et al. showed a significant increase in cancer with lymphoma being the third highest in prevalence [8]. They also had five-year follow-up data that showed significantly more cancer development over this time. Also, Daskalogiannaki et al. in their review of patients with consecutive CT scans found a significantly higher prevalence of cancer in patients with SM compared to those without SM [3]. Besides cancer, SM is associated with other conditions that have been suggested as possible causes, such as previous abdominal surgery or trauma, autoimmune disease, abdominal aortic aneurysm, and infection, but at this time the etiology is unknown [1-3].

SM has a variety of clinical manifestations. The most common presenting manifestations are abdominal pain, nausea, vomiting, altered bowel habits, anorexia, and weight loss many of which were present in our patient [1]. These symptoms are non-specific and suggest many things as possible differential diagnoses including irritable bowel syndrome. However, anorexia and weight loss would point more towards a neoplastic etiology. In addition, multiple asymptomatic cases have been also reported [3]. These cases were diagnosed incidentally after undergoing radiologic workup for other indications. A physical exam can show abdominal tenderness and masses [2].

CT scan is the most convenient diagnostic tool and multiple recent studies use radiological criteria for the diagnosis of SM [4,8,9]. The most sensitive imaging tool for detecting this disease is a dual-phase CT scan with two specific findings, a “fat ring or halo sign” representing a solid fatty mass in the mesentery with nodules and a “tumor pseudocapsule” representing a dense strip of soft tissue surrounding the inflamed mesentery [2]. A misty mesentery was seen in our patient and although this is a consistent CT finding in SM, it is not as specific as the previously described two signs and can be seen in other diseases such as neoplastic infiltration of the mesentery and portal hypertension [6,9]. Although MRI findings are similar to CT, a combination of both may have an advantage as MRI has characteristic findings in the different histopathologic lesions of SM [10]. FDG PET scan could be helpful in excluding neoplasia as SM does not have increased uptake of the tracer [6,9]. Fat necrosis with lipid-laden macrophages (mesenteric lipodystrophy or initial stage), chronic inflammation with lymphocytic infiltrates (mesenteric panniculitis), and fibrosis (retractile mesenteritis or end-stage disease) can be seen on biopsy and may not represent true stages of the disease as they can be present simultaneously [1,11].

Blood biochemistry is usually normal, but the C-reactive protein level and the erythrocyte sedimentation rate may be elevated [1,2]. Kipfer et al. [7] in their series of 53 surgical patients with SM reported three patterns of lesions: type I with diffuse mesenteric thickening, type II with a single discrete mass, and type III with multiple mass lesions.

There is no general agreement regarding the treatment of SM, including medical therapy, surgical therapy, or both together. Asymptomatic patients can be managed without medication. However, the treatment for symptomatic patients is usually individualized and empiric. Spontaneous resolution frequently occurs in untreated patients [1]. Corticosteroids resulted in improvement in some symptomatic patients [1,12,13]. In addition to corticosteroids, colchicine, tamoxifen, 6-mercaptopurine, antibiotics, azathioprine, methotrexate, and infliximab, can be beneficial, however, these suggestions are from a case series so the medical management of SM remains unclear [2]. Surgical resection of the lesion is sometimes difficult due to the existence of associated vascular compromise and the extent of the disease. However, surgery is required for complications like perforation and vascular or intestinal obstruction [2].

## Conclusions

In conclusion, SM is a rare disease that may present similarly to irritable bowel syndrome with abdominal pain and diarrhea or constipation which may cause a delay in diagnosis. A heightened awareness of this disease may prompt further evaluation, especially in patients with more severe or uncontrolled symptoms. Although our patient developed melanoma and lymphoma after her diagnosis of SM, the association between SM and malignancy is not clear. Clinicians should perform a thorough history and physical examination with a detailed skin exam at diagnosis of SM and update all cancer screening tests. Serial CT scans of the abdomen and pelvis with or without MRI could be considered in patients with symptomatic SM. Suspicious lesions should undergo further evaluation with FDG PET and biopsy.
